# Binding Action and Emotion in Social Understanding

**DOI:** 10.1371/journal.pone.0054091

**Published:** 2013-01-17

**Authors:** Francesca Ferri, Sjoerd J. H. Ebisch, Marcello Costantini, Anatolia Salone, Giampiero Arciero, Viridiana Mazzola, Filippo Maria Ferro, Gian Luca Romani, Vittorio Gallese

**Affiliations:** 1 Department of Neuroscience, University of Parma, Parma, Italy; 2 ITAB - Institute for Advanced Biomedical Technologies, Foundation University “G. d’Annunzio”, Chieti, Italy; 3 Department of Neuroscience and Imaging, University “G. d’Annunzio”, Chieti-Pescara, Italy; 4 Institute of Psychiatry, University “G. d’Annunzio”, Chieti-Pescara, Italy; 5 IPRA - Istituto di Psicologia e Psicoterapia Post Razionalista, Roma, Italy; 6 Brain Center for Social and Motor Cognition, Italian Institute of Technology, Parma, Italy; University of Bologna, Italy

## Abstract

In social life actions are tightly linked with emotions. The integration of affective- and action-related information has to be considered as a fundamental component of appropriate social understanding. The present functional magnetic resonance imaging study aimed at investigating whether an emotion (Happiness, Anger or Neutral) dynamically expressed by an observed agent modulates brain activity underlying the perception of his grasping action. As control stimuli, participants observed the same agent either only expressing an emotion or only performing a grasping action. Our results showed that the observation of an action embedded in an emotional context (agent’s facial expression), compared with the observation of the same action embedded in a neutral context, elicits higher neural response at the level of motor frontal cortices, temporal and occipital cortices, bilaterally. Particularly, the dynamic facial expression of anger modulates the re-enactment of a motor representation of the observed action. This is supported by the evidence that observing actions embedded in the context of anger, but not happiness, compared with a neutral context, elicits stronger activity in the bilateral pre-central gyrus and inferior frontal gyrus, besides the pre-supplementary motor area, a region playing a central role in motor control. Angry faces not only seem to modulate the simulation of actions, but may also trigger motor reaction. These findings suggest that emotions exert a modulatory role on action observation in different cortical areas involved in action processing.

## Introduction

Emotions play an important role in shaping social interchange. During everyday social situations actions are tightly linked with emotions. Indeed, motor behaviours are frequently characterized by emotional colouring rather than being mechanically performed, and emotions, manifest in body movements [1], are often motivators of actions [2], [3]. Empirical evidence for the link between action and emotion has been provided by numerous behavioural and neurophysiological investigations. For example, it has been shown that Transcranial Magnetic Stimulation (TMS) induced motor evoked potentials are larger while observing pleasant and unpleasant compared to neutral images [4]. In the same vein, corticospinal excitability facilitation during action observation is higher in amplitude following the presentation of negative than positive stimuli [5]. Differently, imitative tendency seems to be increased specifically by negative emotional stimuli [6]. Finally, there is evidence that emotional states influence also the execution of future movements [7]. All these studies provide evidence supporting the link between emotions and motor behaviour. More precisely, they demonstrate that emotional stimuli affect motor responses as well as action processing. The present study aims at investigating whether and how the emotional context in which an action is embedded, or, more specifically, the emotion expressed by an observed agent, modulates the brain activity underlying the perception of a goal-related action. What is the relevance of such investigation?

Imagine to enter a room and see the guy sitting at the table in front of you grasping a bottle. How can you guess what he is going to do? Whether he is going to drink, because thirsty, or going to throw the bottle towards the door you have just opened, because he has got notice of dismissal from work? His facial expression revealing his emotional state may cue the intention behind his action. In other words, the integration of emotion- and action-related information may facilitate the recognition of the emotional intention of an observed action and, consequently, trigger an appropriate reaction.

At present, the neural mechanisms underlying action and emotional state understanding have been extensively, but separately studied [8], [9], [10], [11], [12], [13], [14], [15], [16], [17], [18], [19], [20], [21], [22]. However, given the tight link between action and emotion, not only the recognition of the former or the latter needs to be investigated, but also or rather their combination.

There is by now large consensus on attributing changes in neural activity produced by the observation of others’ behaviour to an action observation-execution matching mechanism. This has typically been interpreted in the context of a “Mirror Mechanism” (MM). In humans, the MM has been shown to characterize the activation of the lower part of the pre-central gyrus, the posterior part of the inferior frontal gyrus, the rostral part of the inferior parietal lobe, and regions within the intraparietal sulcus. It has been proposed that the MM underpins the shared representation of one’s own executed actions and others’ observed actions; for reviews see [23], [24], [25]. In addition to this “core” motor MM, an emotional cortical circuit endowed with similar mirror properties has been proposed, comprising additional brain regions, such as the insula and adjacent frontal operculum, subserving the representation of emotional bodily states [26], [27], [28]; for a review see [29], [30].

It has been recently argued that specific Embodied Simulation (ES) processes, instantiated by means of MMs (MM-driven ES, [31]) might play a constitutive role in mind-reading, meaning that “people reuse their own mental states or processes in functionally attributing them to others, where the extent and reliability of such reuse and functional attribution depend on the simulator’s bodily resources and their being shared with the target’s bodily resources” [31]. Although MM-driven ES has been so far studied in the domains of action, emotion and sensation separately, it is likely that a given mental state or process (e.g., anger) can be simulated in parallel across several of these domains (e.g., at the sensory-motor and visceromotor level). How can these domains be integrated?

The present study addresses this issue focusing on the modulatory role of emotions on action observation within cortical regions involved in the processing of action- and emotion- related information. Previous studies seem to suggest that some brain regions, such as the pre-central gyrus, the superior temporal sulcus and the insula are actually recruited while separately observing both hand and face actions cueing the agent’s affective state and shaping an emotional context (e.g., [8], [10]). However, as far as we are aware, it is still unknown how such emotional context modulates neural activity related to action processing.

By means of functional Magnetic Resonance Imaging (fMRI), we investigated whether the observation of the same grasping action, either embedded in a context that cues the emotional state of the agent (positive or negative) or in absence of a contextual emotional cue elicits the same or differential neural activity. The effect of emotional context on action observation was tested by means of both a region of interest analysis and voxel-wise contrasts.

## Materials and Methods

### Participants

Twenty-two healthy young adults (8 female, mean age: 27.6 years; range: 21–35), all right-handed [32]; (handedness index >0.8), participated in the present study. All participants had normal or corrected-to-normal vision (correction <0.75) and were naïve as to the purposes of the experiment. Participants gave their written informed consent to participate in the study and were paid for their participation. The study was approved by the Ethics Committee of the “G. d’Annunzio” University, Chieti, and was conducted in accordance with the ethical standards of the 1964 Declaration of Helsinki.

### fMRI Data Acquisition

All images were collected with a 1.5 T Philips Achieva scanner operating at the Institute of Advanced Biomedical Technologies (I.T.A.B. Fondazione “G. d’Annunzio”, Chieti, Italy). Functional images were acquired with a gradient echo EPI sequence. Each subject underwent four scans, each including 216 consecutive volumes comprising 26 consecutive ascending 4-mm-thick slices oriented parallel to the anterior-posterior commissure and covering the whole brain (TR = 2.4 s, TE = 50 ms, 64×64 image matrix, 4×4 mm in-plane resolution; FOV = 256 mm, no gap). A high-resolution structural image was acquired at the end of the session via a 3D MPRAGE sequence (170 sagittal slices, voxel size: 1.25×1.25×1.20 mm, TR = 8.6 ms, TE = 4.0 ms, 192×192 image matrix, FOV = 240 mm).

### Stimuli and Conditions

The experimental stimuli consisted of three sets of coloured movies: 1) “Emotion-Action” (EA), showing an actor (torso, face and arms of either a male or a female) grasping one of four different objects (bottle, pencil case, receiver or CD case placed on a table) with the right hand and facially expressing anger, happiness or no emotion; 2) “Emotion” (E) showing only the face of the actor (either a male or a female) expressing anger, happiness or no emotion; and 3) “Action” (A), showing only the hand action (the field of view was such that the face did not appear). Hence, the experiment comprised the following 7 conditions: 1a) Angry Action (AA, the actor grasped an object expressing anger); 1b) Happy Action (HA, the actor grasped an object expressing happiness); 1c) Neutral Action (NA, the actor grasped an object with a neutral facial expression); 2a) Angry Face (AF, expression of anger); 2b) Happy Face (HF, expression of happiness); 2c) Neutral Face (NF, a face expressing no emotion); 3) Action (A, one of the objects being grasped). All the Emotion conditions were dynamic. The actors in the video clips were seen from a frontal point of view. Actors and different type of objects were presented in equal proportions. Two professional actors, a female and a male, were enrolled as models for the videos (Created by VM and GA). The kinematics of all the presented hand actions was identical in order to avoid that the action emotional context could be inferred by hand kinematics. To obtain such identity, we applied the Blue Screen technique, that is, a technique for compositing two images or frames together in which a color (or a small color range) from one image is removed (made transparent), revealing another image behind it. It was applied to our stimuli in order to superimpose on the same trunk different dynamic facial expressions.

### Design and Procedure

**Figure 1:**
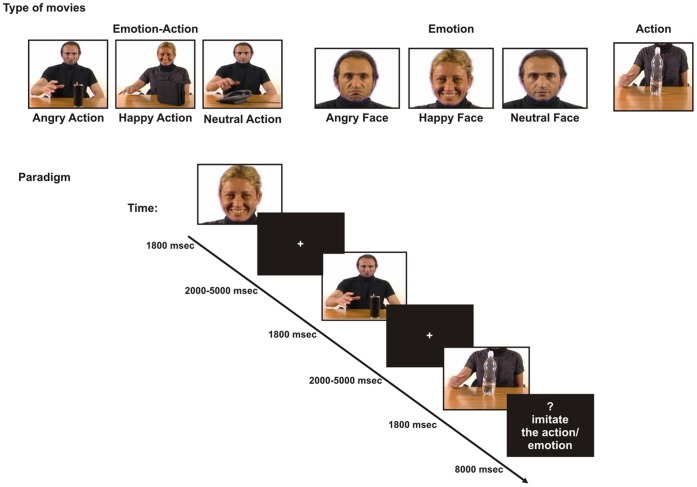
Stimuli and experimental paradigm. Examples of the three sets of coloured movies used in the visual stimulation. Lower panel: description of the experimental paradigm.

The rapid event-related fMRI paradigm consisted of four scans. In each scan 12 movies were presented for each of the seven experimental conditions (AA, HA, NA, A, AF, HF, NF). Each movie lasted 1800 ms and was preceded by a randomized, non-predictable intertrial interval ranging from 2000 to 5000 ms during which a black fixation cross was presented in the centre of a white screen (see figure 1). Participants were instructed to carefully watch the whole scene. To make sure participants paid attention to the experimental stimuli, 8 control trials were randomly inserted in the video sequence of each scan. These unpredictable trials were followed by a question mark lasting 2000 ms followed by a written request (6000 ms) to imitate either the action (4 trials) or the emotion (4 trials) (see figure 1). In total, our experiment consisted of 336 passive observation trials (48 for each experimental condition) and 32 imitation trials (16 for actions and 16 for emotions), presented in pseudo-randomized order.

Subjects lay supine in the scanner with the arms outstretched beside the abdomen. Visual stimuli were projected onto a back-projection screen situated behind the subject’s head and were visible in a mirror (10×15 cm). Sound-attenuating headphones were used to muffle scanner noise. Participants were instructed to carefully watch the whole scene.

### Data Analysis

Functional MRI data were preprocessed and analysed using SPM8 (Wellcome Department of Cognitive Neurology, Institute of Neurology, London). For each subject, functional images were first spatially corrected for head movements using a least-squares approach and six-parameters rigid body spatial transformations [33]. The realigned functional images were then corrected for difference in timing between slices, using the middle slice acquired in time as a reference. The high-resolution anatomical image and the functional images were coregistered and then stereotactically normalized to the Montreal Neurological Institute (MNI) brain template used in SPM8. Functional images were re-sampled with a voxel size of 3×3×3 mm and spatially smoothed with a three-dimensional Gaussian filter of 8 mm full width at half maximum to accommodate anatomical variations between subjects [33]. Images were subsequently analysed using a random-effects approach. At the first stage, the time series of functional MR images obtained from each participant were analysed separately. The effects of the experimental paradigm were estimated on a voxel-by-voxel basis, according to the general linear model extended to allow the analysis of fMRI data as a time series [34]. The onset of each trial constituted a neural event, that was modeled through a canonical hemodynamic response function, chosen to represent the relationship between neuronal activation and blood flow changes [35]. Imitation and question mark period**s** were modelled as separate conditions and then excluded from further analyses.

These single-subject models were used to compute seven contrast images per subject, each representing the estimated amplitude of the hemodynamic response in one of the seven experimental conditions (AA, HA, NA, A, AF, HF, NF), relative to the intertrial baseline. At the second stage, contrast images from all subjects were entered into a full-factorial model, as implemented in SPM8. We first selected regions responding more during at least one of the seven conditions relative to the intertrial baseline. The resulting statistical parametric map of the F statistic was thresholded at p<0.001, corrected for multiple comparisons over the total amount of analysed brain volume using “*Family Wise Error*” (FWE). Based on this map, we created regions of interest (ROIs) for further analyses, by grouping together, for each regional peak, all neighbouring voxels at a maximum distance of 32 mm from the peak.

To localize and visualize the activated clusters we used the BrainShow software [36], [37] implemented in Matlab (MathWorks Inc., MA). The BrainShow software was also used to project group activations onto the standard MNI template and to assign anatomical labels [38].

As a second step, for each identified region, we computed the estimated beta values in each condition (relative to the intertrial baseline), by spatially averaging the pre-processed time series across all voxels in the region and re-estimating the individual general linear models on these averaged time series. Such regional hemodynamic response estimates were then used to perform the following two-tailed simple effect analysis between the Emotion-Action conditions (AA ≠ HA; AA ≠ NA; AA ≠ A; HA ≠ NA; HA ≠ A; NA ≠ A) as well as between the Emotion conditions (AF ≠ HF; AF ≠ NF and HF ≠ NF). According to the Bonferroni correction method the alpha level was set at 0.008 for the contrasts between the Emotion-Action conditions and 0.0167 for the contrasts between the Emotion conditions. Additionally, every single condition was compared with the intertrial baseline by means of one sample t-test.

Finally, the effect of emotional context on action observation was tested by means of whole-brain voxel-wise contrasts. We investigated cortical regions differentiating between observation of actions embedded in emotional contexts as compared to actions embedded in neutral context. To this aim, AA ≠ NA and HA ≠ NA comparisons were performed (p<0.05 FDR). It should be noted that such contrasts also control for low-level visual activations.

## Results

### ROI-based Analysis

**Figure 2:**
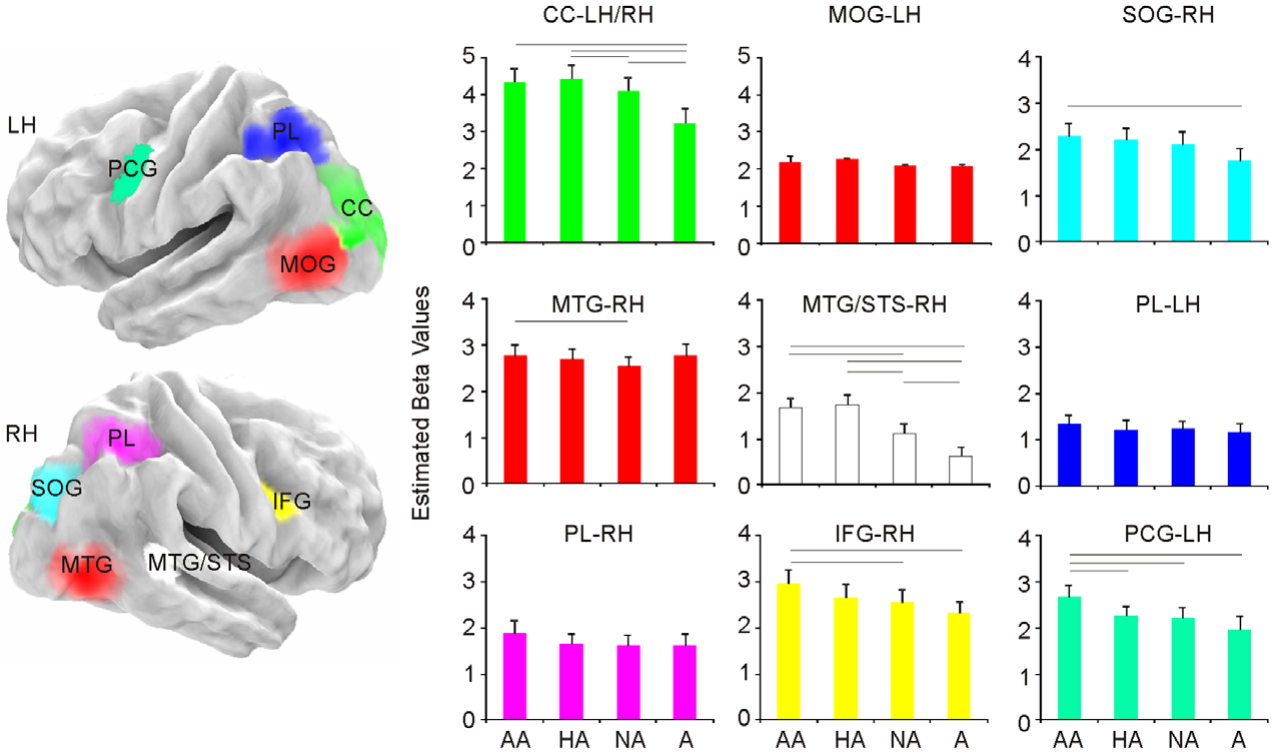
Regions showing different activation during the observation of any experimental condition compared with the intertrial baseline. Group activation data are rendered on the cortical surface of a “canonical” brain (Mazziotta et al., 1995). Plots represent estimated beta values for the AE and A conditions. Vertical bars indicate standard errors. Horizontal bars represent statistical differences.

**Figure 3:**
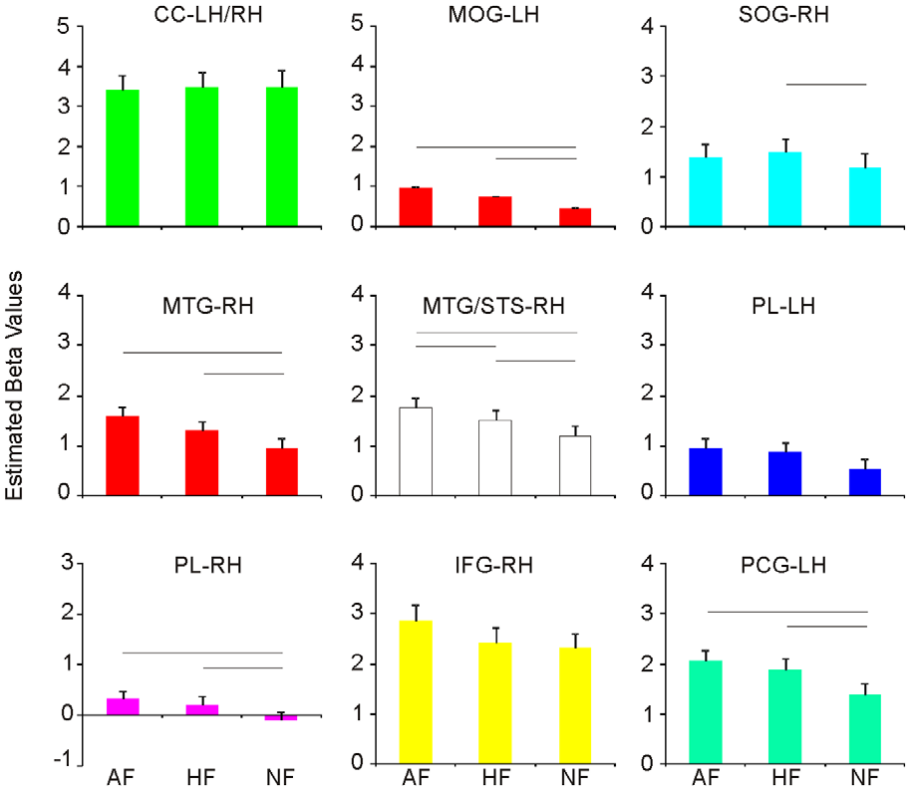
Estimated beta values for the E conditions.

From the group-level whole-brain analysis of functional MR images, with a statistical threshold of p<0.001 (FWE corrected), we identified nine different cortical regions where BOLD signal was significantly higher during at least one of the experimental conditions (AA, HA, NA, A, AF, HF, NF), relative to the intertrial baseline. The nine active regions were located in the left pre-central gyrus (PCG), the right inferior frontal gyrus (IFG), the right temporal cortices, the parietal lobe (PL) bilaterally and the occipital cortex (OC) bilaterally (table 1, figures 2 and 3).

**Table 1:** Montreal Neurological Institute (MNI) coordinates of peaks of relative activation in the cortical regions where BOLD signal was significantly different during observation of any experimental condition compared with the intertrial baseline.

Anatomical region	Side	z-score	Cluster size (voxel)	Main local maxima (MNI)		
				x	y	z
CC	L/R	Inf	1755	9	−81	6
				−3	−102	6
				−24	−84	24
MOG	L	Inf	699	−48	−75	3
SOG	R	Inf	393	24	−84	36
MTG	R	Inf	605	48	−72	−3
	R	Inf	184	54	−39	3
PL	R	Inf	356	−27	−60	51
	L	Inf	344	30	−54	54
IFG	R	7.06	120	48	12	27
PCG	L	Inf	151	−45	3	39

The alfa level was set at 0.001 (FWE Corrected).

*Notes:* CC, calcarine cortex; MOG, middle occipital gyrus; SOG, superior occipital gyrus; MTG, middle temporal gyrus; PL, parietal lobe; IFG, inferior frontal gyrus; PCG, precentral gyrus.

### Left Precentral Gyrus

The cluster centred on the left inferior PCG (48%) extended to the superior PCG (14%) and the IFG (13%). BOLD signal was significantly higher during AA than all the other action conditions (HA, NA, A) [T(21) >3.740; all *p*s >0.001]. Furthermore, activation during observation of both AF and HF was higher than that during observation of NF [T(21) >4.413; all *p*s<0.001] (figures 2 and 3). The BOLD signal levels in this cluster were significantly higher than the intertrial baseline for all the experimental conditions [all *p*s <.05].

### Right Inferior Frontal Gyrus

The cluster centred on the rIFG *pars opercularis* (37%), encompassed the *pars triangularis* (25%) and the PCG (28%). BOLD signal was significantly higher during observation of a grasping action embedded in the emotional context of anger (AA) than in a non emotional context (NA) [T(21) = 3.253; *p*<0.004]. Moreover, BOLD signal was higher during observation of AA compared to A [T(21) = 3.592; *p*<0.002] (figures 2 and 3). The BOLD signal levels in this cluster were significantly higher than the intertrial baseline for all the experimental conditions [all *p*s <.05].

### Right Temporal Cortex

Two activation clusters mapped in the right temporal cortices. The first, centred in Middle Temporal Gyrus (MTG), extended to Middle Occipital Gyrus (MOG), Inferior Occipital Gyrus (IOG) and inferior temporal gyrus (ITG). Average BOLD signal in this cluster was significantly higher during the observation of a grasping action performed by a person expressing anger compared to the same action performed by a person expressing no emotion [T(21) = 3.293; *p*<0.003]. A further modulation of the neural activity in this region resulted for the observation of both AF and HF compared to NF [T(21) >4.502; all *p*s <0.001], and AF versus HF [T(21) = 2.783; *p*<0.011]. The second cluster, centred in MTG, extended to the superior temporal sulcus (MTG/STS). Simple effect analysis of the average regional BOLD response showed that activation in this cluster was significantly higher for the observation of AA, HA and NA compared to A [T(21) >5.086; *p*<0.001 always] and for the observation of a grasping action performed by a person expressing either anger or happiness (AA, HA) compared to the same action performed by a person expressing no emotion (NA) [T(21) = 5.245; *p*<0.001]. A further modulation of the neural activity in this region resulted for the observation of both AF and HF compared to NF [T(21) >3.630; all *p*s <0.002] (figures 2 and 3). The BOLD signal levels in this cluster were significantly higher than the intertrial baseline for all the experimental conditions [all *p*s <.05].

### Left Parietal Lobe

The cluster was centred on left SPL with 73% of the voxels falling within this area. Other voxels fell within the IPL (22%). Activation due to the observation of action embedded in non emotional/emotional context and action alone did not differ from each other (figures 2 and 3). The BOLD signal levels in this cluster were significantly higher than the intertrial baseline for the Action and all Emotion-Action conditions [all *p*s <.05], but not for the Emotion conditions [all *p*s >.05 for AF, HF and NF].

### Right Parietal Lobe

The cluster’s peak value was centred on the right superior parietal lobe (SPL) with 49% of the voxels falling within this region, 23% of the voxels falling within the inferior parietal lobe (IPL) and 13% within the angular gyrus (table 1). BOLD signal in this cluster was significantly higher during the observation of AF [T(21) = 3.116; *p*<0.005] and HF [T(21) = 3.065; *p*<0.006] faces compared to NF. Activation due to the observation of action embedded in non emotional/emotional context and action alone did not differ from each other (figures 2 and 3). The BOLD signal levels in this cluster were significantly higher than the intertrial baseline for all the experimental conditions [all *p*s <.05].

### Occipital Cortex

The activation clusters in the occipital cortex were centred in the calcarine cortex (CC), the left middle occipital gyrus (MOG) and the right superior occipital gyrus (SOG). The cluster centred in the CC extended to the left MOG, the left SOG and the right lingual gyrus (LG). Simple effect analysis of the average regional BOLD response showed that activation in this cluster was significantly higher for the observation of AA, HA and NA compared to A [T(21) >3.317; all *p*s <0.003]. The cluster centred in the left MOG extended to the inferior occipital gyrus (IOG) and the middle temporal gyrus (MTG). Simple effect analysis of the average regional BOLD response showed that activation in this cluster was significantly higher for the observation of Actions embedded in emotional context (both AA and HA) compared to NA [T(21) >3.765; all *p*s <0.001]. The cluster centred in the right SOG extended to MOG. Simple effect analysis of the average regional BOLD response showed that activation in this cluster was significantly higher for the observation of AA than A [T(21) >4.024; *p*<0.001] and for the observation of HF than NF [T(21) >2.993; *p*<0.007]. The BOLD signal levels in this cluster were significantly higher than the intertrial baseline for all conditions [all *p*s <.05] (figures 2 and 3). The BOLD signal levels in this cluster were significantly higher than the intertrial baseline for all the experimental conditions [all *p*s <.05].

### Neural Mapping of Observing Angry and Happy Actions Compared to Neutral Actions

**Figure 4:**
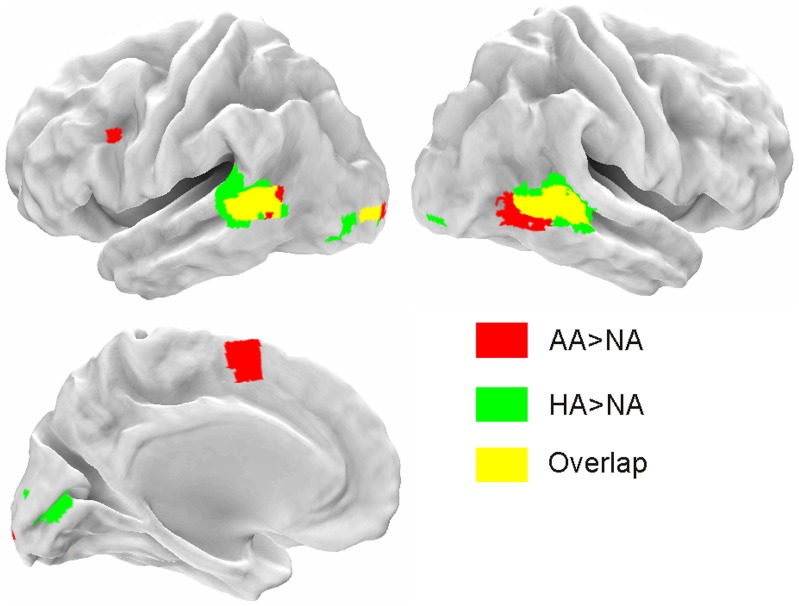
Action-Emotion>Action Neutral. Regions activated either in Angry Action Vs Neutral Action contrast (red) or in Happy Action Vs Neutral Action (green) contrast.

When contrasting the effect of observing either Angry Action or Happy Action with Neutral Action (AA ≠ NA or HA ≠ NA, respectively), the following activation differences were found. Regarding AA relative to NA, higher activation was found in the left IFG (48% pars triangularis and 33% pars opercularis), left pre-SMA, right inferior PCG, MTG/STS bilaterally and left middle OC. Regarding HA, relative to NA, higher activation was found in the MTG/STS, left Fusiform Gyrus (FG), right Lingual Gyrus (LG), middle and inferior OC bilaterally (table 2, figure 4).

**Table 2:** Action-Emotion>Action Neutral.

	Angry Action vs Neutral Action						Happy Action vs Neutral Action				
Anatomical region	Side	Cluster size(voxel)	Main local maxima (MNI)				Cluster size(voxel)	Main local maxima(MNI)			
			x	y	z	z-score		x	y	z	z-score
OC	L/R	20	−24	−99	−3	3.97	18	33	−93	−6	3.52
							226	−3	−84	6	4.13
							47	−33	−78	−9	4.63
LG	R						55	21	−66	−6	4.27
FG	L						24	−36	−63	−12	4.50
MTG/STS	R	168	57	−45	3	4.60	233	54	−45	9	5.28
	L	85	−51	−45	9	5.05	198	−54	−42	9	5.27
PCG	R	13	51	6	39	3.84					
pre-SMA	L	121	−3	6	66	4.35					
IFG	L	21	−36	12	24	3.78					

Local maxima of cortical clusters responding to action embedded in emotional context as compared to actions embedded in neutral context The alfa level was set at 0.05 (FDR Corrected).

*Notes:* OC, occipital cortex; LG, lingual gyrus; FG, fusiform gyrus; MTG, middle temporal gyrus; STS, superior temporal sulcus; PCG, precentral gyrus; pre-SMA, pre-supplementary motor area; IFG, inferior frontal gyrus.

## Discussion

The present study aimed at investigating whether the emotional context, that is, an emotion dynamically expressed by an observed agent, modulates the brain activity underlying the perception of a goal-related action. The experimental conditions included short movies depicting an actor performing a grasping action while expressing an emotion (Happiness, Anger or Neutral), an actor only expressing one of these emotions, or an actor only performing a grasping action (figure 1). These conditions allowed the identification of brain regions sensitive to the modulatory role of emotions on action observation.

### Frontal Cortices

Among cortical frontal areas ventral PCG and IFG, bilaterally, were more activated by observation of actions embedded in the context of anger compared to the observation of actions embedded in a neutral context. Ventral PCG, as well as IFG, are part of a fronto-parietal network critical for action representation [39], [40]. The ventral PCG has been recently proposed to be, at the functional level, the human counterpart of monkey area F5 [41], where mirror neurons were firstly described [42]. The coordinates of local maxima of the PCG clusters in the present study (−45, 3, 39; 51, 6, 39) are similar to the average coordinates (y = 0, z = 41) of activations reported in fourteen out of twenty-three contrasts, reviewed by Morin and Grezes [41], comparing neuronal response elicited by the observation of an action made by a living being with another visual control stimulus. Based on this evidence, these authors proposed that the ventral PCG shares the visual properties of “mirror” neurons found in area F5 of the macaque brain.

Imaging data in humans suggest a role of PCG and IFG in coding the goal of the action [43] and, from another perspective, in the understanding of the agent’s motor intention, driven by the context in which the action is embedded [13]. The present results expand current knowledge by showing that PCG and IFG likely bind the motor information about grasping with the emotional information extracted from the agent’s face, in order to code the motor goal of the action. This holds for anger, but not for happiness (figures 2 and 4). This result suggests that the angry context is combined with the motor representation of the observed action, likely contributing to the immediate ascription of the emotional intention associated with it [44]. As a consequence, it might be hypothesized that this triggers an immediate interaction/re(-en)action from the observer. This hypothesis is supported by the modulated activity in pre-SMA while observing angry actions compared to the observation of neutral actions (figure 3).

Pre-SMA plays a central role in the control of motor behaviour. The higher activation for Angry than for Neutral Action can be interpreted in the light of the role of pre-SMA in the shaping of self-initiated reactions [45]. More specifically, a possible interpretation is that the negative emotional context connoted the perceived action as potentially threatening and, hence, triggered a reaction in the observer. Oliveri and colleagues [45] tested a similar hypothesis by means of a TMS study. They delivered single-pulse TMS over the left primary motor cortex (M1), after a conditioning stimulation of the left SMA, while participants carried out movements in response to pictures with negative or neutral emotional content. Results showed that conditioning of SMA by means of TMS selectively enhanced M1 excitability during the execution of movements triggered by visual cues with negative emotional content, but not by visual-neutral cues. Our results are in line with this finding and with evidence provided by a meta-analysis [46] showing that the SMA/pre-SMA complex is involved in different cognitive functions including attention to one’s own action and valuation of other people’s behaviour.

Alternatively, one may argue that activations in pre-SMA recruitment is related to overt or covered shifts of attention, especially in the angry action condition, as it is more salient than happy and neutral action. Although we agree that angry action attracts more attention than the other conditions, we believe that attentional shifting might not entirely account for our activations. Indeed, if it was the case it remains to be explained why the very same areas are activated also by the observation of action alone and emotion alone, regardless of emotional valence.

### Parietal Cortices

The right and the left parietal cortices, despite being both engaged in hand action’s perception, were not affected by the emotional context. This could be related to the fact the both clusters were centred on and mostly extended within SPL. Data from action imitation and observation studies demonstrated that while the abstract aspects of the action (such as its goal or intention) are represented in IPL, in addition to IFG, the specific (kinematic) aspects of the action [11], [47] are represented in SPL. The absence of a pattern of modulation depending on the emotional context is consistent with this previous knowledge. In fact, during the observation of hand actions performed in different emotional contexts, but with identical kinematics, contextual differences should not affect the representation of the kinematic aspects of the action mapping in SPL. Viewing dynamic face movements led to significant increases in the BOLD signal only in the right parietal cortex, where both angry and happy faces elicited a stronger activity than neutral faces. These results complement also previous data about the body parts specificity of the parietal cortices in the processing of human actions [10], [48] by adding information related to the effect of the emotional context in which the action is performed.

### Other Brain Regions

Finally, regions in bilateral posterior temporal cortex, largely overlapping with MTG/STS, and in left occipital cortex showed increased activation during observation of action embedded in an emotional context, produced by both angry and happy faces. This is in keeping with the data obtained by Wyk and colleagues [49], who proposed that STS is sensitive to the congruency between action and the agent’s intention established by a previous emotional expression. In general, STS is considered to play an important role in the perception of social acts [50]. In particular, right STS seems to be specifically involved in facial emotion recognition rather than in general face processing [51]. As shown in figures 2 and 3, its greater engagement during the emotional conditions might be critical for the emotional modulation of the entire action representation system [8], [10]. In fact, the superior temporal cortex, which is connected to the limbic system via the insula [52], is hypothesized to code an early visual description of the action and to send this information to the action representation network [53], [54].

### Conclusions

The results of the present study suggest that MTG/STS and PCG/IFG, which are part of the action observation system [55], possibly combine action-related information with the specific agent’s affective state. At the level of MTG/STS this occurs regardless of the specific emotional context (i.e. Anger, Happiness). Differently, at the level of PCG/IFG it mainly occurs when the action is performed by angry, but not happy, agents (figure 2 and 4). Selective response for anger in premotor cortex, but not in temporal cortices, has been previously observed comparing brain activations elicited during the perception of anger, threat and neutral behaviours [56]. Even if we did not explicitly assess coding of intentional states, our results might suggest that when viewing actions performed with anger, rather than happiness, an important priority for the brain is to represent the emotion state associated with it.

With our current data, we are not able to disentangle whether the activations in PCG/IFG reveal emotion-related modulations of motor simulation or, alternatively, the preparation of motor response required by the situation. Indeed, coping with angry agents as compared to happy and neutral agents, may require additional behavioral adjustments. In this case, the asymmetry of angry and happy faces would be mainly due to the fact that angry faces lead to stronger arousal boosting activation in movement-related areas, thus, triggering motor reaction. This hypothesis would be supported by the specific effect of anger found in pre-SMA, which plays a central role in the control of motor behaviour (figure 3). Finally, as it is known that negative emotional context facilitates imitative action tendencies [6] and that automatic imitation is mediated by the MM [57], [58], it is also possible that in our study angry faces trigger action imitation, rather than a motor reaction. That said, on the basis of our results, we propose that the modulatory role of emotions on action perception, mainly mapped within PCG/IFG and MTG/STS in the present study, could be viewed as the necessary step towards a more comprehensive social understanding and shaping appropriate social interaction.
